# Adolescents amid the COVID-19 pandemic: a prospective study of psychological functioning

**DOI:** 10.1186/s13034-021-00397-z

**Published:** 2021-08-31

**Authors:** Ieva Daniunaite, Inga Truskauskaite-Kuneviciene, Siri Thoresen, Paulina Zelviene, Evaldas Kazlauskas

**Affiliations:** 1 Center for Psychotraumatology, Institute of Psychology, Vilnius University, Ciurlionio 29, 01300 Vilnius, Lithuania; 2 Norwegian Center for Violence and Traumatic Stress Studies, Oslo, Norway

**Keywords:** COVID-19, Adolescents, Psychosocial functioning, Lithuania

## Abstract

**Background:**

The spread of coronavirus disease (COVID-19) and the accompanying countermeasures can significantly impact the wellbeing of adolescents. There is a lack of longitudinal studies that can shed light on potential social, emotional, and behavioral development in adolescents. We aimed to identify potential changes in adolescent psychosocial functioning from pre-pandemic to peri-pandemic assessment, and secondly, to identify specific patterns of change.

**Methods:**

This longitudinal study was based on a Lithuanian community sample of 331 adolescents aged 12–16 at T1 (*M*  =  13.87, *SD * =  1.59). T1 data collected before the pandemic (March–June, 2019) was compared with T2 data collected during the COVID-19 outbreak (October 2020). Psychosocial functioning was assessed by The Strengths and Difficulties Questionnaire (SDQ). Multivariate latent change modeling and latent class change approaches were used to identify patterns of change.

**Results:**

We found a small but significant increase in hyperactivity/inattention, emotional symptoms, but also prosocial behavior from before to during the pandemic, even adjusting for resilience, lifetime abuse experience, and socio-demographic situation. Three change profiles were identified in the latent change analysis: (1) a majority (70.7%) experienced a significant increase in psychosocial problems; (2) a smaller sub-group (19.6%) with increased peer problems only; (3) a small group (9.7%) showing no negative change and an increase in prosocial behavior.

**Conclusions:**

The study found a significant negative impact of the COVID-19 pandemic on mental health in the majority of adolescents, as well as indications of positive social development in a small group. These findings highlight the importance of identifying and supporting adolescents in the time of the pandemic more effectively. Accumulating knowledge about human responses to the coronavirus, particularly in young people, is pivotal to societal preparedness for future pandemics.

## Introduction

Pandemic diseases may cause major societal disruption and pose great challenges to human adaptation. In recent decades, the likelihood of pandemics has likely increased due to more mobility, urbanization, and other factors [1]. The spread of coronavirus disease (COVID-19) and the accompanying countermeasures can significantly impact the wellbeing of adolescents. Some groups of adolescents might be at a greater risk for serious mental health problems. Previous psychological burden, abuse history, living in a family with low income or low education, belonging to an ethnic minority group are important risk factors for the wellbeing of adolescents during the pandemic [2–6]. In addition, restricted means to psychosocial and social assistance can impact psychosocial functioning [3, 4].

Previous studies showed that adolescents worry about the COVID-19 crisis and are very concerned about their schooling restrictions and peer relationships [7, 8]. Additionally, adolescents reported the negative impact of the COVID-19 pandemic on mental health, learning, friendships, and family relations [6]. Stress, related to the COVID-19 spread and social distancing, was found to be associated with loneliness and depression [7, 9, 10]. Recent studies showed a high level of depression and anxiety in adolescents in different pandemic periods [9, 11–15]. The COVID-19 diagnosis or close contact with an infected person, low social support, and negative coping has been found to relate to higher levels of depression and anxiety [14, 16]. Furthermore, the COVID-19 pandemic seems to have resulted in higher levels of concentration difficulties and restlessness in children and adolescents, as reported by parents [10].

The vast majority of these studies so far have been cross-sectional. A few longitudinal studies show an increase in depression and anxiety symptoms, also the decrease of mental well-being and lower health-related quality of life from before to during the pandemic [8, 17–20]. The highest level of depression and anxiety were associated with peak infection rates, and the decrease of symptoms paralleled the decline in rates of coronavirus [21]. A recent study showed that adolescent mental health trajectories had been altered in the face of COVID-19 [19]. However, not all studies indicate negative changes in mental health in young people due to the pandemic [22]. Thus, we have a very limited understanding of stability and change in adolescent mental health in the context of the COVID-19 pandemic.

The current study aimed to achieve a better understanding of how the COVID-19 pandemic may have affected adolescent mental health and psychosocial functioning in Lithuania. Previous longitudinal research has demonstrated that in terms of adolescent development, Lithuanians are similar to adolescents from other WEIRD (Western, educated, industrialized, rich, and democratic) countries [23]. The COVID-19-related lockdown in the country resulted in closing schools and distant learning, with school lessons being held online for most of the year 2020. Therefore, the contact with teachers and peers was limited to online communication. Additionally, most parents were working from home. This situation introduced new routines when parents and their children spent extensive time at home while also being busy with their tasks. On the one hand, the adolescents experienced a lack of support from other significant adults and peers. On the other hand, handling the study process became one of the additional burdens for parents. These changes might have introduced exposure to new underexplored communication obligations to both parents and their adolescent children. Regarding the lockdown restrictions, the situation in Lithuania in 2020 was similar to other European countries. We assume that due to rapid changes, stress levels have risen and could have affected adolescents’ mental health. Therefore, we first tested if emotional problems, hyperactivity/inattention, behavior problems, and peer problems were higher at 6-months since the first national lockdown during, as compared to before the pandemic. Based on the findings of the previous studies, we hypothesized that adolescent difficulties in emotional and behavioral functioning, problems with peers, and hyperactivity/inattention would be higher than before the pandemic. Second, we sought to identify specific patterns of change. There is substantial evidence that child abuse significantly affects children’s and adolescents’ psychosocial functioning [24–28]. Also, many studies confirmed that psychological resilience mitigates the negative child abuse effect on psychosocial functioning [24, 29–34]. Therefore, we controlled for child abuse experience and psychological resilience in our latent class change analysis in this study.

## Method

### Participants and procedure

This study is based on the data from the first two waves of the ongoing longitudinal study Stress and Resilience in Adolescence (STAR-A). The STAR-A study is implemented by the Center of Psychotraumatology at Vilnius University in Lithuania. The study was approved by the Ethics Committee for Psychological Research in Vilnius University. Information on the procedures of the STAR-A study has been published previously [28, 35].

The current analysis focused on a subsample of 331 adolescents aged 12–16 at the time of the first wave of research, March–May 2019. Data were obtained at two time points: baseline/pre-test (T1, wave 1) and in 18 months at 6 months since the first national lockdown in Lithuania amid the COVID-19 outbreak (T2, wave 2). The T2 data was collected from September 24 to October 21, 2020. During this period, school closing was required at some level [36]. Depending on the COVID-19 situation in the municipality or community, each school could choose the teaching strategy—live, distant, or hybrid. People from outside were not allowed to enter the school premises. Gatherings were restricted to 10 and fewer people [36]. By the start of the data collection on September 24, 2020, there were 9586 identified COVID-19 cases in Lithuania in total, including 116 deaths with a trend of increasing cases and deaths until the end of 2020 [37].

The data for this study were collected in 7 general schools from different regions across Lithuania. Data in the first wave was collected using the paper–pencil method. Data in the second wave was collected online through the platform designed for online surveys. The procedures for the T2 data collection were adapted to the pandemic situation (the second wave of the COVID-19) and valid restrictions in the country. According to the previous adolescent studies, web-based surveys can be applied without the risk of disadvantages compared to paper–pencil assessments [38].

Before starting data collection in 2019, written assent from adolescents and written informed consent from legal guardians were obtained. The protection of study participants’ identities was ensured; randomly generated IDs were assigned for the participants in T1. Information about psychological help possibilities was provided to all study participants in T1 and T2.

In cooperation with schools, 449 students were invited to participate in T2. Most of the adolescents (336, 74.8%) completed the self-report online questionnaire. Responses from five adolescents had to be removed from the analysis because their T1 and T2 data could not be merged due to the lack of identification information provided by the study participant. The final study sample comprised of 331 adolescents, mean age at T1 13.87 (*SD * =  1.59) years, 57.4% girls (*n*  =  190). The majority of participants were born in Lithuania (98.8%, *n * =  327) and were of Lithuanian nationality 92.1% (*n * =  305). More than two-thirds of the sample (71.3%, *n * =  236) were from two-parent families. In terms of demographic characteristics, our study sample was not representative but highly comparable with the general population of adolescents [39]. All demographic characteristics of study participants in T2 are presented in Table 1.

**Table 1:** Characteristics of study participants at T1 (N = 331)

Variable	*n*	%
Gender		
Male	141	42.6
Female	190	57.4
Age		
Mean (SD)	13.87 (1.59)	
Range	12–16	
Family structure		
Two-parent	236	71.3
Other	95	29.7
University education of parents		
One/both of parents	215	64.9
None	27	8.2
Don’t know	89	26.9
Place of birth		
Lithuania	327	98.8
Other	4	0.2
Nationality		
Lithuanian	305	92.1
Other	12	3.7
Missing	14	4.2

### Measures

#### Psychosocial functioning

Psychosocial functioning of adolescents was measured in T1 and T2 using the Strengths and Difficulties Questionnaire (SDQ) [40]. The SDQ includes 25 items, comprising five scales with five items in each. The response format is a 3-point Likert scale. Five psychosocial functioning dimensions are evaluated: emotional symptoms, conduct problems, hyperactivity, peer problems, and prosocial behavior. The SDQ has been previously validated in Lithuania [41, 42]. The SDQ is widely used globally and has shown acceptable reliability and validity across many cultures [43].

#### Resilience

The psychological resilience of adolescents at T1 was measured by The Resilience Scale (RS-14) [44]. The RS-14 scale includes 14 items measuring the construct of psychological resilience. The response format is a 7-point Likert scale. The Lithuanian version of the scale was used and validated in the adult and adolescent populations previously [45, 46].

#### Lifetime abuse exposure

Lifetime abuse exposure was measured in T1 using the questionnaire developed by the Norwegian Center for Violence and Traumatic Stress Studies (NKVTS). The questionnaire included 37 questions covering six types of abuse: neglect at home (6 items), emotional abuse at home (8 items), physical abuse from an adult at home (6 items), online sexual abuse (5 items), sexual abuse from adults (6 items), sexual abuse from peers (6 items). All single items were reported previously [28]. The response format for neglect questions was a 5-point scale ranging from “never” (0) to “very often/always” (4). The participant was considered as exposed to neglect if (s)he responded to any neglect item with “sometimes” (2), “often” (3), or “very often/always” (4). Concerning the items on all other forms of abuse, the participants were asked to respond on a 4-point scale ranging from “never” (0) to “often” (3). The participant was considered as exposed to emotional abuse if (s)he responded to any emotional abuse item with “sometimes” (2) or “often” (3), and physical/sexual abuse—if (s)he responded to any physical/sexual abuse item accordingly with an answer “once” (1), “sometimes” (2) or “often” (3). Finally, the participant was considered as exposed to lifetime abuse, if (s)he has experienced one or more types of abuse.

### Data analysis

To examine the changes in the indicators of adolescent psychosocial functioning at the COVID-19 outbreak (T2), in comparison to pre-test (T1), we used the multivariate latent change modeling approach that provides robust estimates of change over time [47]. In latent change models with two measurement points, the intercept represents the adjusted mean level of the measure at T1, and the slope represents the change from T1 to T2. In the current study, we conducted the latent change model of five parallel processes: change in prosocial behavior, hyperactivity/inattention, emotional symptoms, conduct problems, and peer relationship problems. When running the model, we accounted for possible effects of gender, age, the level of resilience at T1, and the lifetime abuse exposure (exposed vs. not exposed measured a T1) on indicators of psychosocial functioning and included them as control variables by regressing on all intercepts and slopes. To have the latent change model identified, first, we fixed the residuals to zero; second, we fixed non-significant effects of control variables to zero one by one until we obtained the final model with the links of at least marginal significance (*p * <  0.10) only. In addition, to identify whether the change processes in indicators of psychosocial functioning were linked with each other and whether the initial levels of prosocial behavior, hyperactivity/inattention, emotional symptoms, conduct problems, and peer relationship problems were associated with the changes, we correlated all intercepts and slopes. The model fit in latent change analysis was evaluated by using the Comparative Fit Index (CFI), the Tucker–Lewis Index (TLI), and the Root Mean Square Error of Approximation (RMSEA), following the goodness of fit recommendation provided by Kline [48]. Namely, CFI/TLI values higher than 0.90 indicated an acceptable fit, and values higher than 0.95 represented a very good fit; RMSEA values below 0.08 indicated an acceptable fit, and values less than 0.05 suggested a good fit.

After running the multivariate latent change model, we sought to identify groups of participants with possibly different patterns of change in indicators of psychosocial functioning by using the latent class change approach [49]. We classified the study participants based on the change in all five indicators (i.e., prosocial behavior, hyperactivity/inattention, emotional symptoms, conduct problems, and peer relationship problems) with also including the control variables used in latent change analysis. We used several criteria to decide on the number of latent classes. First, the Akaike Information Criterion (AIC) and Bayesian Information Criterion (BIC) statistics for a solution with k classes should be lower than for a solution with k−1 classes. Second, a statistically significant *p *value of the parametric bootstrapped likelihood ratio test, which compares improvement in fit between neighboring class solutions after the inclusion of an additional class. Third, Entropy scores above 0.70 with relatively higher values indicative of more accurate classification. When reporting the change in indicators of psychosocial functioning, the bias-corrected effect sizes [50] were reported. The magnitude of the effect expressed in *d* was interpreted according to Cohen [51], that is, 0.20  =  small effect, 0.50  =  medium effect, and 0.80  =  large effect. The analyses were conducted with Mplus 8.2. [52].

## Results

Means and standard deviations of the study variables at T1 and T2, and correlations among them, are presented in Table 2. The correlations between values of each five indicators of socioemotional functioning at T1 and T2 were significant and moderate to high (0.46–0.63). Prosocial behavior at T1 was significantly negatively associated with hyperactivity/inattention, conduct, and peer problems at T1. Prosocial behavior at T2 was significantly and negatively associated with concurrent hyperactivity/inattention and conduct problems, but significantly and positively with concurrent emotional difficulties.

**Table 2:** Correlations among study variables at the baseline (T1) and COVID-19 outbreak (T2)

		M (SD)	γ_1/_γ_2_	1	2	3	4	5	6	7	8	9	10
1	Prosocial behavior T1	7.14 (2.00)	− 0.55/− 0.16	1									
2	Hyperactivity/inattention T1	3.39 (2.07)	0.43/− 0.27	− 0.247***	1								
3	Emotional symptoms T1	2.86 (2.29)	0.72/− 0.17	0.039	0.308***	1							
4	Conduct problems T1	2.53 (1.53)	0.75/0.52	− 0.209***	0.400***	0.183**	1						
5	Peer relationship problems T1	2.27 (1.84)	0.97/0.63	− 0.250***	0.148**	0.335***	0.207***	1					
6	Resilience T1	56.11 (10.18)	− 0.44/0.08	0.301***	− 0.334***	− 0.418***	− 0.216***	− 0.290***	1				
7	Prosocial behavior T2	6.94 (2.12)	− 0.66/0.30	0.528***	− 0.152**	− 0.002	− 0.092	− 0.196***	0.095	1			
8	Hyperactivity/inattention T2	3.84 (2.24)	0.36/− 0.22	− 0.109*	0.568***	0.205***	0.295***	0.020	− 0.243***	− 0.107	1		
9	Emotional symptoms T2	3.27 (2.47)	0.51/− 0.61	0.045	0.209***	0.626***	0.120*	0.169**	− 0.312***	0.117*	0.290***	1	
10	Conduct problems T2	2.49 (1.57)	0.75/0.43	− 0.115*	0.287***	0.168**	0.488***	0.167**	− 0.124*	− 0.152**	0.355***	0.200***	1
11	Peer relationship problems T2	2.33 (1.77)	0.62/− 0.14	− 0.236***	0.162**	0.295***	0.081	0.463***	− 0.241***	− 0.274***	0.087	0.370***	0.213***

*M* mean; *SD* standard deviation; *γ*_1_ skewness; *γ*_2_ kurtosis

^*^p  <  0.05; **p  <  0.01; ***p  <  0.001

### Change in mental health indicators

**Fig. 1:**
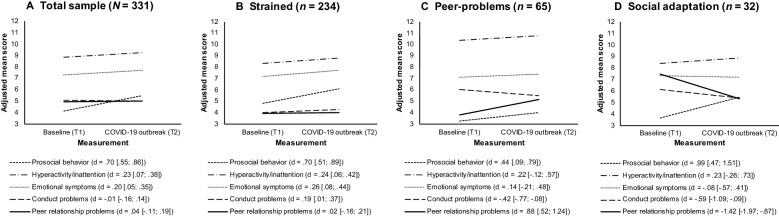
The trajectories of change in psychosocial functioning indicators in **A** total sample, **B** strained, **C** peer-problems, and **D** social adaptation latent change classes (N  =  331). *d*  effect size with 95% confidence intervals. A negative score indicates a decrease, a positive score indicates an increase

The multivariate latent change analysis yielded an excellent model fit [χ^2^ (26)  =  26.95, *p*  =  0.412, CFI/TLI  =  0.999/0.997, RMSEA (0% CI)  =  0.010 (0.000, 0.045), SRMR  =  0.029]. Overall, we found a significant but small increase in rates of hyperactivity/inattention (*M*_slope_  =  0.45, *p*  <  0.001) and emotional symptoms (*M*_slope_  =  0.41, *p * <  0.001), as well as large increase in prosocial behavior (*M*_slope_  =  1.32, *p * =  0.034) from T1 to T2 with no change in conduct problems (*M*_slope_  =  − 0.02, *p * =  0.852), and peer relationship problems (*M*_slope_  =  0.07, *p * =  0.495). For all indicators of psychosocial functioning, we found significant negative links (*p * <  0.001) between intercepts and slopes, indicating that lower baseline rates of prosocial behavior (*r * =  − 0.39), hyperactivity/inattention (*r * =  − 0.41), emotional symptoms (*r * =  − 0.39), conduct problems (*r * =  − 0.48), and peer relationship problems (*r * =  − 0.52) were associated with bigger increase in these indicators. The trajectories of change in the indicators of psychosocial functioning in a full study sample are presented in Fig. 1.

We found significant gender effects on intercepts of prosocial behavior (*β*_intercept_  =  − 0.31, *p*  <  0.001) and emotional symptoms (*β*_intercept_  =  − 0.25, *p* < 0.001), indicating higher baseline rates of these indicators in girls, compared to boys. Also, the results showed significant negative age effects on intercepts of hyperactivity/inattention (*β*_intercept_  =  − 0.10, *p * =  0.019) and conduct problems (*β*_intercept_  =  − 0.12, *p * =  0.011), indicating the higher rates of these indicators at the baseline being associated with younger age. A higher resilience level at T1 was found to be positively linked with the intercept of prosocial behavior (*β*_intercept_  =  0.32, *p * <  0.001) and negatively linked to the intercepts of hyperactivity/inattention (*β*_intercept_  =  − 0.31, *p * <  0.001), emotional symptoms (*β*_intercept_  =  − 0.35, *p * <  0.001), conduct problems (*β*_intercept_  =  − 0.15, *p * =  0.002), indicating that higher resilience was associated with better psychosocial functioning at baseline. Also, we found significant abuse exposure effects on intercepts of emotional symptoms (*β*_intercept_  =  0.12, *p * =  0.002), indicating higher levels of internalizing problems at the baseline in the abuse exposure group, compared to the non-exposure group. Finally, we found that the slope of prosocial behavior was significantly negatively linked with resilience (*β*_intercept_  =  − 0.18, *p * <  0.001) and positively linked with abuse exposure (*β*_intercept_  =  0.09, *p*  =  0.045), indicating that the bigger increase in prosocial behavior was observed in adolescents with lower baseline rates of resilience as well as in the abuse exposure group, compared to the non-exposure group.

### Patterns of change in psychosocial functioning

The latent class change analysis indicated that the three classes solution fitted the data best (see Table 3). Three identified change profiles were found to be clearly distinguishable in terms of differences in changes of psychosocial functioning indicator means over time. Most adolescents (70.7%) reported a significant but small increase in hyperactivity/inattention (*M*_slope_  =  0.45, *p*  =  0.005), emotional symptoms (*M*_slope_  =  0.53, *p * <  0.001), and conduct problems (*M*_slope_  =  0.26, *p * =  0.040) with a stability in prosocial behavior (*M*_slope_  =  1.26, *p * =  0.051) and peer relationship problems (*M*_slope_  =  0.04, *p * =  0.754); we labeled this pattern as strained. Almost one in five adolescents (19.6%) reported large increase in Peer relationship problems (*M*_slope_  =  1.36, *p*  <  0.001) with no significant change in other indicators of psychosocial functioning: prosocial behavior (*M*_slope_  =  0.78, *p*  =  0.259); hyperactivity/inattention (*M*_slope_  =  0.43, *p * =  0.369), emotional symptoms (*M*_slope_  =  0.29, *p*  =  0.411), and conduct problems (*M*_slope_  =  − 0.56, *p * =  0.085); we labeled this pattern as peer-problems. Finally, almost one in ten adolescents (9.7%) reported a large increase in prosocial behavior (*M*_slope_  =  1.81, *p * =  0.014) and a large decrease in peer relationship problems (*M*_slope_  =  − 12.19, *p * <  0.001) with a stability in hyperactivity/inattention (*M*_slope_  =  0.45, *p*  =  0.356), emotional symptoms (*M*_slope_  =  − 0.16, *p * =  0.741), and conduct problems (*M*_slope_  =  − 0.79, *p*  =  0.059); we labeled this pattern as social adaptation. The trajectories of change in the indicators of psychosocial functioning in three classes are presented in Fig. 1B, C, D. The peer-problems group was characterized by high scores on hyperactivity at both time points. The social adaptation group was characterized by high scores on peer problems at T1.

**Table 3:** Model fit indices of latent class change analyses

Solution	Loglikelihood	AIC	BIC	Entropy	BLRT *p*-value	Smallest class count (%)
1 class	− 6333.05	12,824.10	13,124.47	–	–	–
2 classes	− 6333.05	12,846.10	13,188.29	< 0.001	1.000	50.00
**3 classes**	**− 6275.53**	**12,753.07**	**13,137.08**	**0.859**	**0.000**	**9.67**
4 classes	− 6260.74	12,745.48	13,171.32	0.811	0.030	11.78

The best fitting solution is in bold

*AIC* Akaike Information Criterion; *BIC* Bayesian Information Criterion; *BLRT* Parametric Bootstrapped Likelihood Ratio

## Discussion

In this two-wave longitudinal study, we investigated the changes in adolescents’ psychosocial functioning amid the COVID-19 pandemic in contrast to pre-pandemic functioning, exploring mental health changes at 6-month since the onset of the first lockdown. In Lithuania, the COVID-19 countermeasures included the closure of schools and restrictions on other essential areas of social life for adolescents in the country. At T2, the schools were just partly reopened. Overall, we found a small but significant increase in hyperactivity/inattention, emotional symptoms, and prosocial behavior. At the same time, the rates of conduct problems and peer problems did not change significantly in the total sample. Our findings align with the results of several previous longitudinal studies, which found higher levels of depression and anxiety among adolescents amid the pandemic [2, 6, 8, 17, 18, 20]. However, not all previous studies indicated the negative changes in adolescents’ mental health related to the pandemic [22]. In line with the previous studies [53–55], emotional problems and prosocial behavior at the baseline were higher in girls than boys. As lower baseline rates of emotional problems were associated with a bigger increase, boys may have suffered a bigger increase in emotional problems amid the pandemic. Overall, our results highlight that adolescents in the general population experience psychosocial difficulties during the pandemic, which might constitute a risk for future mental health problems.

Analysis of specific patterns of change in adolescents’ psychosocial functioning revealed three different change profiles of adolescents’ psychosocial functioning compared to before the pandemic. This analysis gives us insights into specific challenges different groups of adolescents can meet during this or future pandemics. Based on this analysis, scientists and practitioners can plan different specific prevention and intervention strategies. Around two-thirds of the sample had a small but significant increase in hyperactivity/inattention, emotional symptoms, and conduct problems (strained group). These changes can be related to pandemic stress and life changes, loneliness, social isolation, distant learning-related concentration difficulties, or lack of motivation [5, 9, 10, 12, 56]. General prevention strategies, helping to organize the learning environment and daily tasks, emotional support and stress management strategies, can be helpful for the majority of adolescents and young people. These could include the online adaptation of prevention and socio-emotional skills training programs, discussions, groups activities, and social gatherings online.

According to our results, one in five adolescents experienced an increased peer problems (peer-problems group) compared to before the pandemic. The previous studies show that increased peer problems can be associated with social restrictions, social isolation, less time with friends, and less perceived friend support [9, 17]. The analysis showed that this subgroup had a high level of hyperactivity or attention problems before the pandemic. Adolescents with hyperactivity and attention problems can have more difficulties adapting to the changing conditions and keeping social contacts online. Peer relationship and hyperactivity/inattention problems are serious risk factors for later mental health disorders [2, 57]. The results indicate that parents, teachers, and other school personnel should pay particular attention to the social relationships in this group and foster positive ways of online communication. The previous studies show that the core elements of adolescent friendships persist in online communication [58]. Further research is, however, necessary to replicate this finding in other contexts and samples.

Finally, our study shows that almost one in ten adolescents reported a significant decrease in peer relationship problems and an increase in prosocial behavior (social adaptation group) with the stability in other indicators compared to before the pandemic. This group of adolescents, who experienced a relatively high level of peer problems before the pandemic, showed a substantial decrease in peer problems during the pandemic. An increase of prosocial behavior in the COVID-19 pandemic context and after other stressful events was already documented in the previous studies and can be recognized as a positive adaptation [59, 60]. Reduction of peer problems might be associated with the previous difficulties with peers in school, such as bullying. We speculate that these adolescents might have difficulties when they return to school, and need to be recognized and supported by professionals. The results show that such stressful situations as pandemics and lockdown can release prosocial communication opportunities for some adolescents. School staff can use these findings by promoting volunteering and positive interactions in creative ways.

The changes in adolescents’ psychosocial functioning can be associated with the circumstances related to the pandemics—school closures, restrictions on after-school activities, social contacts with relatives and friends. The study results provide insights for prevention and intervention strategies for the pandemic and post-pandemic period, also preparation for possible future disasters and stressors. General prevention strategies for psychosocial difficulties and specific strategies helping adolescents creating safe contacts and maintain friendships are needed in such periods as lockdowns and school closures. Still, some adolescents can be struggling more with daily communication with peers when they come back to direct learning. Future longitudinal research to follow the trajectories of adolescents’ functioning after the pandemic is needed.

## Limitations

The current study has many strengths, including the longitudinal design, high response rate, and inclusion of the pre-pandemic measures to estimate changes in psychosocial functioning during the COVID-19 pandemic. Still, several limitations are to be mentioned. Despite the longitudinal data collection on psychosocial functioning, it is not possible to attribute the detected changes specifically to the effects of the COVID-19 pandemic on mental health. The time between the two measures of 18 months is a relatively long period in an adolescent’s life, and many changes in family and peer life can happen during such time. Moreover, the data were based on self-report and more objective assessments of psychosocial functioning, and reports from parents and teachers could address this limitation in future studies. Finally, the data were collected in one European country with a relatively homogenous sample in a high-income country. It cannot be ensured that the results are generalizable to all the countries, especially having different COVID-19 rates and variable government response measures across countries, more heterogeneous, and migrant populations.

## Conclusions

This study contributes to the existing literature by showing a decline in the psychosocial functioning of adolescents as a potential consequence of the pandemic. Our findings highlight the importance of prevention and intervention measures to help adolescents cope with psychosocial challenges related to pandemics or similar highly stressful situations in the future. Changes in psychosocial functioning can serve as an antecedent of later mental health problems. Peer relations in the context of social restrictions and after returning to school require special attention, and fostering the adolescents’ prosocial behavior can act as an important protective factor. Moreover, parents and professionals should be capable of monitoring the psychosocial functioning of adolescents and provide the needed support, according to the specific challenges adolescents meet. Prevention measures of mental health problems in adolescence, responding to the pandemic-related challenges, and returning to usual daily life routine challenges, are needed.

## Data Availability

The datasets generated and analyzed during the current study are not publicly available due to ethical reasons but are available from the corresponding author on reasonable request.

## Abbreviations

COVID-19: Coronavirus disease of 2019
SDQ: The Strengths and Difficulties Questionnaire
RS-14: The Resilience Scale
CFI: Comparative Fit Index
TLI: The Tucker–Lewis Index
RMSEA: The Root Mean Square Error of Approximation
